# DNA barcodes successfully identified Macaronesian *Lotus* (Leguminosae) species within early diverged lineages of Cape Verde and mainland Africa

**DOI:** 10.1093/aobpla/plu050

**Published:** 2014-08-21

**Authors:** Dario I. Ojeda, Arnoldo Santos-Guerra, Felicia Oliva-Tejera, Ruth Jaen-Molina, Juli Caujapé-Castells, Águedo Marrero-Rodríguez, Quentin Cronk

**Affiliations:** 1 The Biodiversity Research Centre, Department of Botany, University of British Columbia, 6804 SW Marine Drive, Vancouver V6T 1Z4, Canada; 2 Unidad de Botánica-ICIA, Puerto de la Cruz, Canary Islands, Tenerife, Spain; 3 Jardín Botánico Canario ‘Viera y Clavijo'–Unidad Asociada CSIC, Cabildo de Gran Canaria Las Palmas, Gran Canaria, Spain

**Keywords:** Conservation, DNA barcoding, island radiation, *Lotus*, Macaronesia, species identification.

## Abstract

Plant DNA barcoding currently relies on the application of a two-locus combination, *matK* + *rbcL*. Despite the universality of these two gene regions across plants, it is suspected that this combination might not have sufficient variation to discriminate closely related species. In this study, we tested the performance of this two-locus plant barcode along with the additional plastid regions *trnH*-*psbA*, *rpoC1* and *rpoB* and the nuclear region internal transcribed spacer (nrITS) in a group of 38 species of *Lotus* from the Macaronesian region. The group has radiated into the five archipelagos within this region from mid-Miocene to early Pleistocene, and thus provides both early divergent and recent radiations that pose a particularly difficult challenge for barcoding. The group also has 10 species considered under different levels of conservation concern. We found different levels of species discrimination depending on the age of the lineages. We obtained 100 % of the species identification from mainland Africa and Cape Verde when all six regions were combined. These lineages radiated >4.5 Mya; however, in the most recent radiations from the end of the Pliocene to the mid-Pleistocene (3.5–1.5 Mya), only 30 % of the species were identified. Of the regions examined, the intergenic region *trnH-psbA* was the most variable and had the greatest discriminatory power (18 %) of the plastid regions when analysed alone. The nrITS region was the best region when analysed alone with a discriminatory power of 26 % of the species. Overall, we identified 52 % of the species and 30 % of the endangered or threatened species within this group when all six regions were combined. Our results are consistent with those of other studies that indicate that additional approaches to barcoding will be needed in recently evolved groups, such as the inclusion of faster evolving regions from the nuclear genome.

## Abstract (precis)

Plant barcoding uses short DNA sequences to identify unknown samples at species level. This technique relies on the universality of these gene regions and the existence of enough variation among species to allow discrimination. Island radiations pose one challenging scenario where insufficient variation has accumulated in recently diverged groups to allow species identification. In this work we tested whether six gene regions are suitable for barcoding such a radiation in the Macaronesian *Lotus*. We found high levels of species discrimination in lineages of 3.5 Mya old or older and that the efficiency drastically reduces for younger radiations.

## Introduction

DNA barcoding is a procedure that uses universal DNA sequences to assign species names to sampled individuals (http://www.barcodeoflife.org/). Plant DNA barcoding is currently performed with the two-locus (*matK* and *rbcL*) recommended by the CBOL Plant Working Group (2009). This suggested combination is able to discriminate ∼72 % of the samples used by the CBOL Plant Working Group at the species level, with the remaining samples assigned to congeneric species groups.

Many of the studies that have tested regions as barcodes in plants have focused on large data sets that span a wide range of land plants, or at least angiosperms (Kress and Erickson 2007; Fazekas *et al.* 2008; Lahaye *et al.* 2008; CBOL Plant Working Group 2009; Ford *et al.* 2009). Their purpose has been the assessment of the universal applicability of the regions in species discrimination. However, it has been argued that the success in species discrimination of DNA barcodes will drop in (i) some groups with complex biology and (ii) closely related species within the same genus (or in recently evolved groups) (Pillon *et al.* 2013).

To date, the level of species discrimination within the same genus has been tested in a number of cases (Sass *et al.* 2007; Newmaster *et al.* 2008; Newmaster and Ragupathy 2009; Song *et al.* 2009; Starr *et al.* 2009; Clerc-Balin *et al.* 2010; Liu *et al.* 2010; Wang *et al.* 2010), and at least some groups of closely related species will be problematic for barcoding (Sass *et al.* 2007; Miller *et al.* 2009; Seberg and Petersen 2009). When individual genera are sampled more extensively, the percentage of species discrimination tends to decrease, even when several regions are combined (Kondo *et al.* 2007; Sass *et al.* 2007; Edwards *et al.* 2008; Seberg and Petersen 2009).

The Macaronesian *Lotus* has colonized and radiated into the five volcanic archipelagos (Azores, Madeira, Salvage Islands, Canary Islands and Cape Verde) within this region from mainland Africa (Allan *et al.* 2004; Ojeda *et al.* 2012). This group comprises ∼41 described species, divided into two sections: *Pedrosia* and *Rhyncholotus* (or the ‘rhyncholotus group’) (Degtjareva *et al.* 2006). Section *Pedrosia* comprises 37 recognized species, while section *Rhyncholotus* comprises only four species (Allan *et al.* 2004). The two groups are distinguished by contrasting floral morphology associated with different pollination syndromes, but within each group vegetative features are more useful for species recognition and identification (Sandral *et al.* 2006). Based on these vegetative and reproductive structures, Sandral *et al.* (2006) further subdivided this group into nine informal taxonomic groups (Table 1), which reflects the lineages recovered in the most recent phylogenetic analysis (Ojeda *et al.* 2012). Many of these species are restricted to specific habitats, such as the pine forest and the lowland scrub. Furthermore, ∼70 % of them are endemic to single islands. Thus, the group is highly susceptible to habitat destruction, and at least 10 species are listed under some category of conservation threat, ranging from rare to critically endangered (VV.AA. 2000; Martín *et al.* 2008; Bañares *et al.* 2011; Table 2).

**Table 1.** Dates of divergence from the MCRA in the eight informal taxonomic groups within Pedrosia and the Rhyncholotus group. *Taxonomic groups following morphological features according to Sandral *et al.* (2006). The remaining groups classified in this study. ^¶^Species not sampled in this analysis.

Informal taxonomic groups below section level	Species	Distribution	Age of divergence (Mya)	Identification success (%)
*L. purpureus* group	*L. arborescens*	Cape Verde	4.78	100
	*L. bollei*			
	*L. brunneri*			
	*L. jacobaeus*			
	*L. purpureus*			
	*L. latifolius*			
*L. jolyi* group*	*L. jolyi*	Africa		
	*L. tibesticus^¶^*			
*L. arenarius* group*	*L. arenarius*	Africa, Spain	4.55	100
	*L. maroccanus*			
	*L. eriosolen*			
*L. sessilifolius* group*	*L. sessilifolius*	Canary Islands	3.71	30
	*L. mascäensis*			
	*L. arinagensis*			
	*L. emeroides*			
	*L. kunkelii*			
*Rhyncholotus* group*	*L. berthelotii*			
	*L. eremiticus*			
	*L. maculatus*			
	*L. pyranthus*			
*L. argyrodes* group*	*L. argyrodes*	Azores, Madeira	2.5	33
	*L. macranthus*			
	*L. azoricus*			
	*L. loweanus^¶^*			
*L. campylocladus* group*	*L. callis-viridis*	Canary Islands	4.34	40
	*L. campylocladus*			
	*L.* aff. *spartioides*			
	*L. holosericeus*			
	*L. hillebrandii*			
	*L. spartioides*			
	*L. dumetorum*			
*L. glaucus* group*	*L. glaucus*	Canary Islands, Salvage Islands and Madeira		
	*L. tenellus*			
	*L. leptophyllus*			
	*L. salvagensis*			
	*L. lancerottensis*			
	*L. erythrorhyzus*			
*L. assakensis* group*	*L. assakensis*	Africa, Mediterranean		
	*L. creticus*			
	*L. pseudocreticus*			
	*L. chazalei^¶^*

**Table 2.** Macaronesian *Lotus* species considered under different levels of threat, according to Red List of Spanish Vascular Flora based on the IUCN Red Data Book (IUCN) (VV.AA. 2000), the Atlas of Endangered Spanish Vascular Flora (AESVF) (Bañares *et al.* 2011) and the ranking according to the top 100 endangered species of Macaronesia (Martín *et al.* 2008). Numbers indicate their rank under the top 100 lists, –, not considered within the 100 most endangered species. CR, critically endangered; EN, endangered; VU, vulnerable.

Species	Distribution	IUCN 2000	AESVF 2004	Rank within the top 100 in Macaronesia
*L. arinagensis*	Canary Islands	CR	CR	–
*L. berthelotii*	Canary Islands	CR	CR	7
*L. callis-viridis*	Canary Islands	EN	EN	–
*L. dumetorum*	Canary Islands	VU	–	–
*L. eremiticus*	Canary Islands	CR	CR	25
*L.* aff. *spartioides*	Canary Islands	–	CR	–
*L. kunkelli*	Canary Islands	CR	CR	6
*L. maculatus*	Canary Islands	CR	CR	3
*L. mascaensis*	Canary Islands	VU	–	–
*L. pyranthus*	Canary Islands	CR	CR	–
*L. spartioides*	Canary Islands	–	VU	–

The applicability of the recommended barcode regions for species recognition within very recently evolved groups, such as those resulting from island radiations, has not being extensively tested (Mort *et al.* 2010; Pillon *et al.* 2013). It is unclear whether the levels of DNA variation observed in the two-locus recommended barcodes of the CBOL Plant Working Group will allow species discrimination in groups that radiated on islands. Here we present the assessment of five plastid regions suggested as barcodes in previous studies (*matK, rpoC1, rpoB, trnH-psbA* and *rbcL*) and the nuclear ribosomal internal transcribed spacer (ITS) as barcodes within the Macaronesian *Lotus*. Additionally, we related the performance of species identification of these six barcodes with age estimates of each lineage and the time of most recent common ancestor (MRCA) of each lineage within each archipelago and mainland Africa.

In this study, we aim to address the following questions: (i) Are these six DNA regions (*matK*, *rpoC1*, *rpoB*, *trnH-psbA*, *rbcL* and ITS) variable enough to allow species discrimination within the different age lineages in the Macaronesian *Lotus* group? (ii) Are these six barcodes variable enough to allow species discrimination for the endangered species of this group? and (iii) Can these regions reliably identify assemblages (informal taxonomic groups) of species recognized on morphological grounds?

## Methods

### Taxon sampling

Our sampling included 78 accessions representing all the species currently described within the sections *Pedrosia* and *Rhyncholotus*, except for three species (*Lotus loweanus*, *L. chazalei* and *L. tibesticus*) that were not available for this analysis. For 10 species we were unable to add more than one sample to represent the species and we included more than one accession for 27 species. Our analysis also included accessions from some populations that (based on previous molecular and morphological analyses) may represent four new undescribed species within the section *Pedrosia* (Oliva-Tejera *et al.* 2005, 2006; Sandral *et al.* 2006; A. Santos-Guerra, Unidad de Botanica-ICIA, pers. comm.). For comparison, we also included five accessions from section *Lotus* **[see** **Supporting Information****]**.

### Dating the phylogeny of Macaronesian *Lotus*

The Macaronesian *Lotus* seems to have colonized this region from mainland Africa more than once (Allan *et al.* 2004) and the ornithophilous traits present in the four species of the *Ryncholotus* group evolved recently, within the last 2 Mya (Ojeda *et al.* 2012) from a group of entomophilous ancestors (Ojeda *et al.* 2013). The group has colonized the five volcanic archipelagos of this region at different times and it has recent species radiations in some of these archipelagos (e.g. Canary Islands) (Ojeda *et al.* 2012). The most recent phylogenetic analysis of the group recovered four major clades using a combined analysis of four nuclear (ITS, three *CYCLOIDEA* homologues) and two chloroplast (*trnH-psbA* and *matK*) regions (Ojeda *et al.* 2012). Despite the nearly complete sampling of the group and the number of gene regions used, the most recently diverged clades had moderate-to-low bootstrap support. In order to obtain an estimate of the divergence times of the nine informal taxonomic groups identified within the Macaronesian *Lotus*, we used a combined matrix of 52 samples and four gene regions (ITS, *matK*, *trnH-psbA* and *CYB6*) with a total of 2092 bp. Divergence times were obtained using the program Beast v1.5.4 (Drummond and Rambaut 2007), and the analysis was done using a constant-rate Yule (speciation process) prior and all other priors and operators with the default settings. Four independent runs were performed using the uncorrelated lognormal relaxed-clock model (Drummond *et al.* 2006) for 50 000 000 generations. Trees and parameters were sampled every 5000 generations, yielding a total of 10 000 trees, with a burn-in of 5 000 000. All analyses were run using the HYK + gamma substitution model. The Beast file was created using the BEAUti program v 1.5.4 within Beast. The performance of each run was further analysed with the program Tracer. Mean parameter estimates and 95 % highest posterior densities were determined by analysing the Beast tree files with TreeAnnotator v 1.5.4 (Drummond and Rambaut 2007). Trees were visualized and edited with Figtree v1.3.1. This analysis was constrained with the best hypothesis of relationship (topology) of this group obtained from MP and ML (Ojeda *et al.* 2012).

The topology was calibrated in three points. Two points were calibrated using two endemic taxa from two different islands, *Lotus sessilifolius* subsp. *villossisimus* (El Hierro, 1.12 Mya) and *L. sessilifolius* subsp. *sessilifolius* (La Palma 1.77 Mya) (Ancochea *et al.* 1994; Carracedo 1994). The third calibration point of 20.6 Mya was based on the age of the oldest island, Fuerteventura, as an upper limit for the colonization of the Canary Islands (Carracedo 1994) and therefore an upper limit for the age of the MRCA for the species of this archipelago.

### Barcode regions selected

We sequenced six regions: the recommended two-locus cpDNA barcode (*matK* *+* *rbcL*; CBOL Plant Working Group 2009), three other cpDNA regions (*trnH*-*psbA*, *rpoB* and *rpoC1*) and the nuclear ITS region, which has been assessed in some plant groups as a barcode (Chase *et al.* 2005; Kress *et al.* 2005; Kress and Erickson 2007).

### Molecular analysis

Genomic DNA was extracted from fresh leaves, silica-gel dried leaf material or voucher specimens following standard procedures (Doyle and Doyle 1987). Amplification was carried out with the following PCR conditions for all the plastid regions: 94 °C for 3 min, 30 cycles of 94 °C for 3 min, 45 °C for 1 min and 72 °C for 2 min, with a final cycle of 72 °C for 5 min. The nuclear ribosomal intergenic spacer ITS was amplified using the following conditions: 94 °C for 3 min, 30 cycles of 94 °C for 1 min, 55 °C for 1 min and 72 °C for 1.5 min, with a final cycle of 72 °C for 5 min. Each locus was sequenced and the raw sequence data were imported to Sequencher 4.1 for editing and constructing contig sequences. Consensus sequences were imported to Se-Al ver. 1.0 (Rambaut 1996). To eliminate sequencing error, sequence quality was carefully assessed. Polymorphisms were sequenced on both strands and dubious cases re-sequenced. Each region was analysed separately and the accessions with failed amplifications were removed.

### Assessment of the barcode regions

Three requirements have been suggested for the official barcodes: universality, sequence quality and coverage, and discrimination (CBOL Plant Working Group 2009). We evaluated these three parameters in the six regions tested within this group.

*Universality*: we estimated the percentage of amplification success on the first trial as an indicator of universality, using the same PCR conditions.

*Sequence quality and coverage*: we estimated the percentage of bidirectional sequences with few or no ambiguous bases for each region.

*Discrimination*: we evaluated discrimination at two levels: species discrimination and discrimination of informal taxonomic groups following previous taxonomic analysis based on morphological features (Sandral *et al.* 2006). Nine informal taxonomic groups at the infrageneric level have been suggested within the Macaronesian assemblage (Table 1). We considered that useful discrimination at this level was achieved when at least 50 % of the species were assigned within the same group. For species discrimination we used a distance-based method to assign species. Each region was analysed separately and in various combinations with neighbour-joining (NJ) using Kimura two-parameter as the standard in barcoding applications. We also analysed the data using unweighted pair group method with arithmetic mean and parsimony (Lahaye *et al.* 2008) as implemented in PAUP4b10 (Swofford 2001). However, those methods did not result in any major differences in species discrimination. We also tested whether the inclusion or exclusion of missing sequences affected species discrimination in two-locus combinations and when all five plastid regions were combined with ITS.

## Results

**Figure 1.**
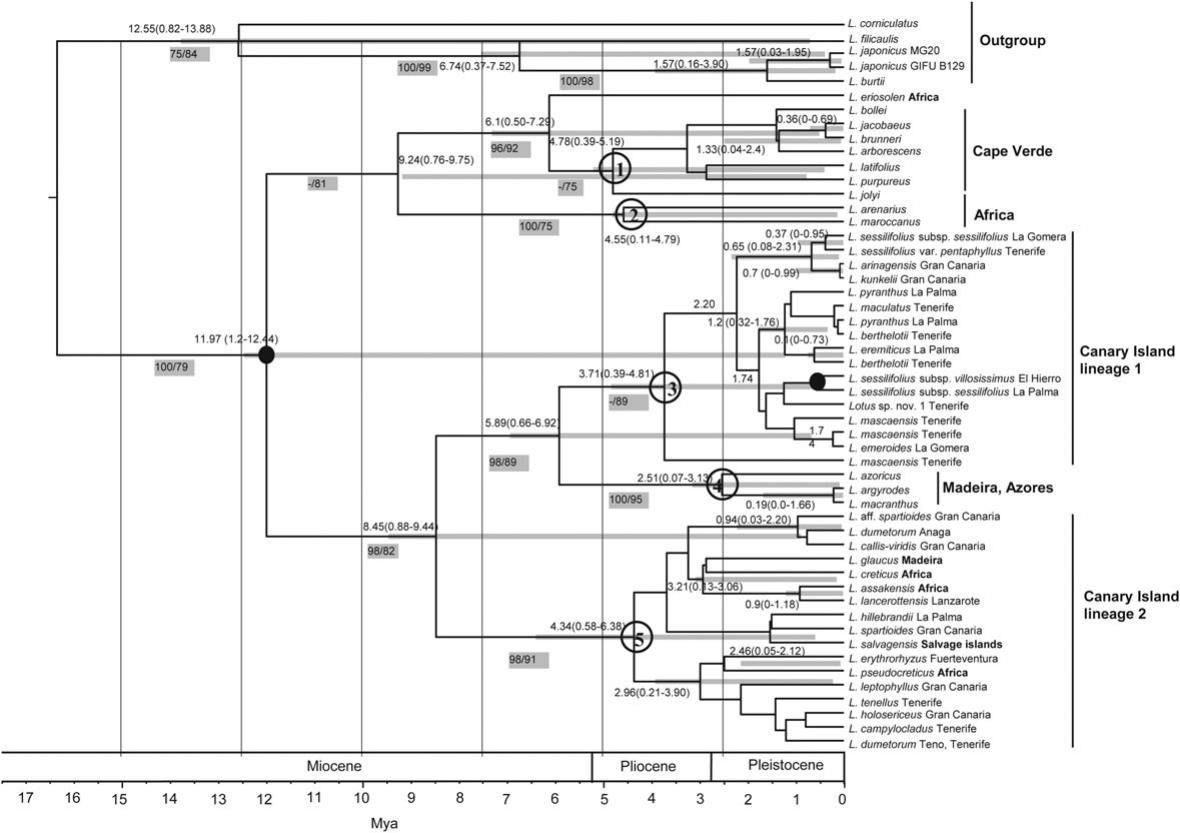
Chronogram obtained for the divergence of MRCA of the nine informal taxonomic groups within *Pedrosia* and *Rhyncholotus* (number in circles). The following informal taxonomic groups were considered: (1) *L. purpureus*/*L. jolyi* group, (2) *L. arenarius* group, (3) *L. sessilifolius*/*Rhyncholotus* group, (4) *L. argyrodes* group and (5) *L. campylocladus*/*L. glaucus*/*L. assakensis* group. The tree was calibrated using a data set of 52 samples and a data set of four gene regions (ITS, *matK*, *trnH-psbA* and *CYB6*) (Ojeda *et al.* 2012) under a Bayesian relaxed-clock, uncorrelated clock model using BEAST. Upper limits of the ages of La Palma (1.77 Ma), El Hierro (1.12 Ma) and Fuerteventura (20.6 Ma) were used as calibration points (black circles). Age estimates with their 95 % credibility intervals are shown on nodes. Values in grey squares represent bootstrap values from MP/posterior probabilities inferred from the Bayesian inference.

We found that the barcode regions tested successfully identified early diverged species from Cape Verde and mainland Africa and Europe but the success was reduced in more recent speciation events. The different lineages included within Macaronesian *Lotus* diverged and radiated within this archipelago at different times. The earliest divergent lineages include two African groups (*Lotus arenarius* and *L. jolyi*) and the lineage that colonized Cape Verde (*Lotus purpureus*). All the species included within these three groups had 100 % of species discrimination when all five regions were combined, and even when individual regions were analysed alone (Table 3 and Fig. 1). Species discrimination was greatly reduced on the lineages that diverged at the end of the Pliocene and beginning of the Pleistocene (3.5 to 2 Mya) within the Canary Islands, Madeira and the Salvages.

**Table 3.** Performance of the five plastid regions tested separately and in some combinations with the nuclear ITS gene region. *Informal sections according to Sandral *et al.* (2006). A, including all accessions; B, excluding accessions with missing sequences in two-pair combinations.

	Aligned sequence (bp)	No. of species discriminated: total/endangered		No. of informal taxonomic groups discriminated*	
One region					
ITS	621	10/0		3	
*trnH-psbA*	342	7/1		4	
*matK*	867	7/1		4	
*rpoC1*	511	5/1		0	
*rbcL*	588	2/0		0	
*rpoB*	354	0/0		0	
Plastid combinations		A	B	A	B
*matK* + *trnH-psbA*	1209	11/2	13/2	4	4
*matK* + *rpoC1*	1378	10/2	10/3	4	3
*rpoC1* + *trnH-psbA*	853	10/1	9/0	3	3
*rbcL* + *trnH-psbA*	930	7/1	9/1	3	3
*matK* + *rbcL*	1455	7/1	7/1	3	2
*matK* + *rpoB*	1221	6/1	6/0	3	2
*rpoB* + *trnH-psbA*	696	5/0	6/0	4	4
*rbcL* + *rpoC1*	1099	3/0	3/0	0	0
*rpoB* + *rpoC1*	865	5/1	5/1	0	0
*rbcL* + *rpoB*	942	4/0	3/0	1	0
All plastids combined	2662	9/2	14/3	4	4
ITS + plastid					
ITS + *trnH-psbA*	963	15/3	14/3	4	4
ITS + *rpoC1*	1132	12/1	11/1	3	3
ITS + *matK*	1468	11/1	7/0	4	4
ITS + *rpoB*	975	11/1	11/1	3	3
ITS + *rbcL*	1209	10/0	9/0	3	3
All six regions combined	3283	19/4	17/3	4	4

All regions had >95 % sequencing success, except for the *matK* region with 83 % success, due to failure of amplification or due to regions with T or A repeats that caused failure during sequencing. This region had the lowest level of bidirectional sequence quality (Table 4).

**Table 4.** The six gene regions tested in this analysis with their specific primers and performance.

Region	Primer pair	PCR success	Sequencing success	No. of indels	Parsimony informative sites
*trnH-psbA*	Fw PA	96	98	2	13
	Rev TH				
*matK*	matK2.1F	83	85	0	24
	matK3.2X				
*rpoC1*	rpoC1F	96	100	0	9
	rpoC14R				
*rbcL*	80F	97	100	0	19
	ajf634R1				
*rpoB*	rpoB2F	97	100	0	5
	rpoB3R				
ITS	ITS4	100	99	2	69
	ITS5				

**Figure 2.**
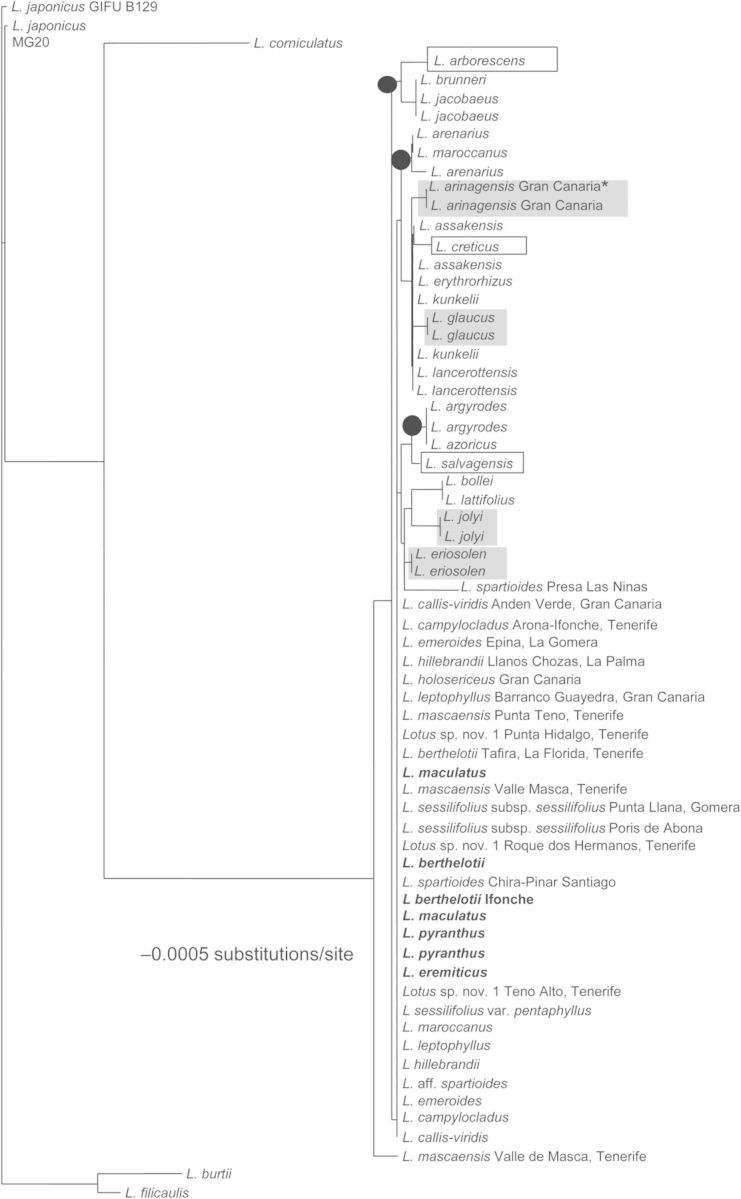
Neighbour-joining tree generated with the combination of the CBOL recommended two-locus, *matK* + *rbcL*. Grey squares represent species with more than one sample and species in a square represent species with a single accession. Branches with black circles represent informal taxonomic groups identified. Species in bold belong to section *Rhyncholotus* while species not in bold are included within section *Pedrosia.* Asterisks indicate endangered species successfully barcoded.

The *trnH-psbA* and *rpoB* regions showed the highest and the lowest level of variation and species discrimination of all regions evaluated, respectively (Table 3). The combination *trnH-psbA* + *matK* showed the highest level (34 %) of discriminatory power at the species level for two-locus combinations. Three two-locus combinations (*trnH-psbA* + *matK*, *matK* + *rpoC1*, *rpoC1* + *trnH-psbA*) showed slightly better discriminatory power than the barcode recommended by CBOL (*matK* + *rbcL*) (Fig. 2 and Table 3).

When all five plastid regions were combined, we achieved the identification of 14 species (36 %) of the 38 species in our sample (Table 3). Even using five regions only 3 of the 10 species (30 %) of conservation concern were identified at the species level.

We were able to identify only four informal taxonomic groups with the combination of *matK* + *trnH-psbA* and no improvement was observed when all regions were combined, or with any region when analysed alone (Table 3). The intergenic spacer *trnH-psbA* was the only plastid region in which we observed intraspecific variation, due to two indels and a small inversion.

**Figure 3.**
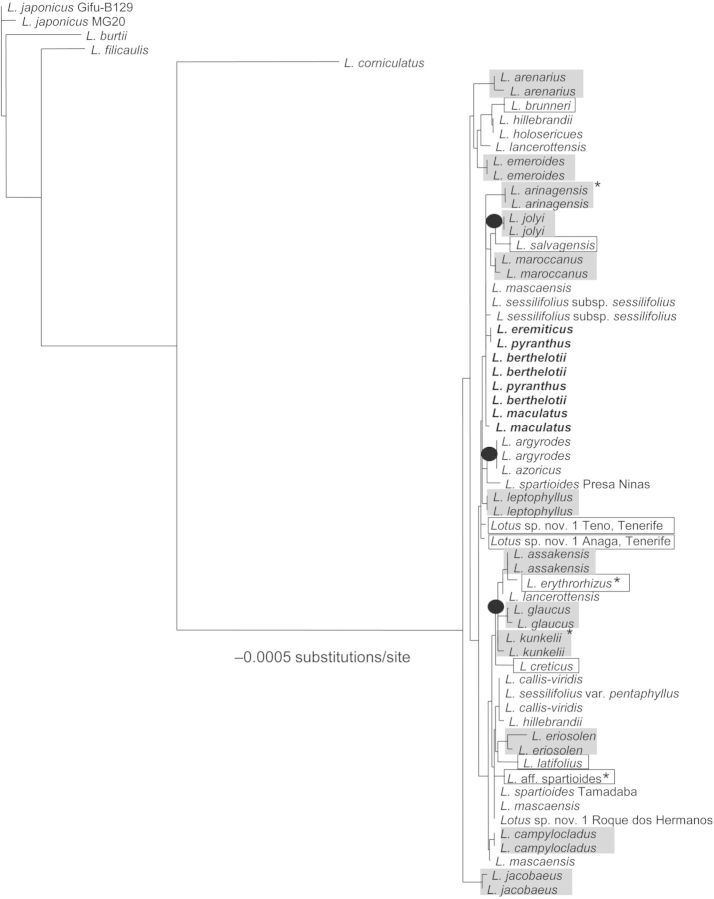
Neighbour-joining tree generated with the combination of all six regions tested (*rbcL, matK, trnH-psbA, rpoC1, rpoB*, and the nuclear ribosomal ITS). Grey squares represent species with more than one sample and species in a square represent species with a single accession. Branches with black circles represent informal taxonomic groups identified. Species in bold belong to section *Rhyncholotus* while species not in bold are included within section *Pedrosia.* Asterisks indicate endangered species successfully barcoded.

The ITS region showed the highest level of variability of all regions tested in this study when analysed alone, with a species identification rate of 26 %. The overall level of species discrimination increased substantially when we combined this region with a plastid region, with the best two combinations being ITS + *trnH-psbA* and ITS + *matK* (Table 3). The addition of ITS increased the discriminatory power in the species sampled overall, with 52 % species discrimination when all six regions were combined (Fig. 3). However, even with six regions we were able to identify only 30 % of the species of conservation concern.

## Discussion

Plant DNA barcoding of phylogenetically diverse assemblages has proven successful with high levels of species discrimination, e.g. Panamanian trees with 98 % of species identification (Kress *et al.* 2009) and Mesoamerican orchids with >90 % of species identification (Lahaye *et al.* 2008), but the success of species discrimination tends to decrease as the number of species within families or genera is increased (Gonzalez *et al.* 2009; Xiang *et al.* 2011; Yesson *et al.* 2011; Zhang *et al.* 2011; Arca *et al.* 2012; Maia *et al.* 2012; Saarela *et al.* 2013). Previous studies have reported relatively low (55 % using *trnH-psbA* in *Aspalathus*) to moderately high percentages (e.g. 92 % in *Crocus*) of species discrimination in several congeneric plant groups (Sass *et al.* 2007; Edwards *et al.* 2008; Seberg and Petersen 2009), and it has been suggested that barcodes will have some limitations in closely related species (Chase and Fay 2009), and especially on island radiations (Pillon *et al.* 2013).

In the particular case of the Macaronesian *Lotus,* we were able to identify 18 % of the samples at the species level with the CBOL suggested two-locus combination (*matK* + *rbcL*) (Fig. 3 and Table 3) and only 52 % of the samples when all six regions were combined. This contrasts with the general rate of discrimination success with *matK* + *rbcL*, which is ∼70 % taking as a reference the database used by CBOL (CBOL Plant Working Group 2009). The nrITS region showed the highest level of species discrimination (26 %) of all regions, and *trnH-psbA* (18 %) of the plastid barcodes tested. These two regions have also low levels of variation in the recent lineages of *Lotus*. The *L. sessilifolius* and the *Rhyncholotus* groups have identical ITS sequences, despite the differences in vegetative and floral traits between the two groups (Ojeda *et al.* 2012).

Although the percentage of species discrimination within the Macaronesian *Lotus* is low overall, the discriminatory power of these barcode regions is not uniformly distributed across the lineages we analysed. Early divergent groups within Africa and Europe (*Lotus jolyi* and *L. arenarius* group in Fig. 1) have 100 % of species discrimination. The same applies for lineages that presumably colonized this archipelago early (*L. purpureus* group, Fig. 1).

The successful application of barcodes in recent radiations will depend on several factors, including the colonization time and the time of the most recent radiations within each particular group. To date, barcodes have been only tested in species of *Tolpis* within Macaronesia (Mort *et al.* 2010) and at the floristic level in 64 endemic taxa from 23 angiosperm families within the Garajonay National Park in La Gomera (Jaén-Molina *et al.* 2010). Using two combinations of four plastid regions (*matK*, *rpoC1*, *rpoB* and *trnH-psbA*), Mort *et al.* (2010) found high levels of species discrimination in the majority of the morphologically distinct species within the genus *Tolpis* (Asteraceae), even within the relatively recent radiated clades of the Canary Islands. Using the two-locus (*matK* + *rbcL*) recommended barcode, Jaén-Molina *et al.* (2010) found similar levels of species discrimination compared with other geographical regions where these two regions have been applied (CBOL Plant Working Group 2009), suggesting that these two regions have practical application in species discrimination in this particular island.

Therefore, it seems that the limited success of species discrimination we found in the Macaronesian *Lotus* might be associated with their recent colonization and diversification. In Hawaii, the recently radiated genera *Cyrtandra* and *Clermontia* also have lower levels of species discrimination within the *rbcL*, *trnH-psbA* and *matK* regions (Pillon *et al.* 2013), suggesting that recent island radiations might pose a difficulty for barcoding. Although the current amount of examples of barcoding island radiations is too limited to draw definitive conclusions, it seems that there is a threshold at which these species are too young to be barcoded with the current markers. It has been suggested, for instance, that the Hawaiian species of *Cyrtandra* and *Clermontia* have a threshold between 3–4.7 and 2–3 Mya, respectively (Pillon *et al.* 2013). In the particular case of the Macaronesian *Lotus*, we found that lineages <3 Mya have reduced species discrimination. Kim *et al.* (2008) identified three windows of colonization within other Macaronesian plant groups. Some groups colonized Macaronesia in the late Miocene (*Aeonium,* 15.2 Mya) or in the late Pliocene (*Sideritis,* 3.3 Mya), but most of them radiated during the Pleistocene (<3 Mya). Those groups that colonized Macaronesia relatively recently, and also those that radiated within the last 3 Mya, might represent a particular challenge to barcodes. Additional studies in other plant groups will be necessary to estimate the overall success of barcodes in this geographical region.

Besides the age of colonization and radiation of this group, the current taxonomy and species delimitation within the Macaronesian *Lotus* group could also explain the overall low levels of species discrimination. In this particular case we used a relatively narrow species concept, but one that follows usual taxonomic practice in the Macaronesian flora (Sandral *et al.* 2006). Further re-assessment of the species boundaries within this group is necessary in order to fully evaluate the effectiveness of barcodes in this group.

## Conclusions

In this study, we found that species discrimination in recent radiations in oceanic islands will be lower compared with continental counterparts. In the particular case of Macaronesian *Lotus*, we have shown that the discriminatory power of the barcodes is not homogeneous in all lineages, and radiations that occurred recently (≤3.5 Mya) will have the lowest levels of species discrimination. Species discrimination was successful in continental African species and lineages that radiated earlier than 4 Mya in this group, but additional approaches will be required for the most recent radiations.

With the reduction of costs in high-throughput next-generation sequencing, the application of ultra-barcoding, or the generation sequencing to produce whole organellar genomes and substantial nuclear ribosomal sequence (Kane *et al.* 2012), is potentially a suitable approach to overcome such rapid radiation in oceanic islands. This approach has been successfully applied to identify subspecies, varieties and individual genotypes in *Theobroma cacao,* and it will be a suitable approach to identify more variable regions in the genome of other plant groups in which evolution occurred on recent island radiations.

## Accession Numbers

All sequences obtained in this study from fresh, herbarium specimens and silica gel preserved material have been deposited in the GenBank data base under the accession numbers KM372590–KM373057.

## Sources of Funding

Funding for this work was provided by Consejo Nacional de Ciencia y Tecnología (CONACyT) from Mexico to D.I.O., by a grant from the NSERC Discovery Grant program from Canada to Q.C. and by the project No. RF2006-00030 from the Spanish Instituto Nacional de Investigación y Tecnología Agraria y Alimentaria to A.S.G.

## Contributions by the Authors

D.I.O. and Q.C. conceived the idea of the paper. D.I.O., A.S.-G., F.O.-T., R.J.-M. and Á.M.-R. participated in collecting plant material in the field and from herbarium specimens; D.I.O. performed the analysis, D.I.O., A.S.-G., R.J.-M., J.C.-C. and Q.C. prepared the manuscript.

## Conflicts of Interest Statement

None declared.

## Supporting Information

The following Supporting Information is available in the online version of this article –

**Table S1.** Species from the sections *Pedrosia* and *Rhyncholotus* sampled in this analysis. Distribution: G, La Gomera; P, La Palma; T, Tenerife; GC, Gran Canaria; CV, Cape Verde; M, Madeira; H, Hierro; L, Lanzarote; F, Fuerteventura; UBC, University of British Columbia; JBCVC, Jardín Botánico Canario ‘Viera y Clavijo’—Unidad Asociada CSIC; JAO, Jardín de Aclimatación de La Orotava.

Additional Information

## References

[PLU050C1] Allan GJ, Francisco-Ortega J, Santos-Guerra A, Boerner E, Zimmer EA (2004). Molecular phylogenetic evidence for the geographic origin and classification of Canary Island *Lotus* (Fabaceae: Loteae). Molecular Phylogenetics and Evolution.

[PLU050C2] Ancochea E, Hernan F, Cendrero A, Cantagrel JM, Fuster JM, Ibarrola E, Coello J (1994). Constructive and destructive episodes in the building of a young oceanic island, La Palma, Canary Island, and genesis of the Caldera de Taburiente. Journal of Volcanology and Geothermal Research.

[PLU050C3] Arca M, Hinsinger DD, Cruaud C, Tillier A, Bousquet J, Frascaria-Lacoste N (2012). Deciduous trees and the application of universal DNA barcodes: a case study on the circumpolar *Fraxinus*. PLoS ONE.

[PLU050C4] Bañares A, Blanca G, Güemes J, Moreno JC, Ortiz S (2011). Atlas y Libro Rojo De La Flora Vascular Amenazada de España.

[PLU050C5] Carracedo JC (1994). The Canary Islands: an example of structural control of the growth of large-oceanic islands volcanoes. Journal of Volcanology and Geothermal Research.

[PLU050C6] CBOL Plant Working Group (2009). A DNA barcode for land plants. Proceedings of the National Academy of Sciences of the USA.

[PLU050C7] Chase MW, Fay MF (2009). Ecology. Barcoding of plants and fungi. Science.

[PLU050C8] Chase MW, Salamin N, Wilkinson M, Dunwell JM, Kesanakurthi RP, Haidar N, Savolainen V (2005). Land plants and DNA barcodes: short-term and long-term goals. Philosophical Transactions of the Royal Society of London. Series B, Biological Sciences.

[PLU050C9] Clerc-Balin JL, Starr JR, Bull RD, Saarela JM (2010). A regional approach to plant DNA barcoding provides high species resolution of sedges (*Carex* and *Kobresia*) in the Canadian Artic Archipelago. Molecular Ecology Resources.

[PLU050C10] Degtjareva GV, Kramina TE, Sokoloff DD, Samigullin TH, Valiejo-Roman CM, Antonov AS (2006). Phylogeny of the genus *Lotus* (Leguminosae, Loteae): evidence from nrITS sequences and morphology. Canadian Journal of Botany.

[PLU050C11] Doyle JJ, Doyle JL (1987). A rapid DNA isolation procedure for small amounts of fresh leaf tissue. Phytochemical Bulletin.

[PLU050C12] Drummond AJ, Rambaut A (2007). BEAST: Bayesian evolutionary analysis by sampling trees. BMC Evolutionary Biology.

[PLU050C13] Drummond AJ, Ho SYW, Phillips MJ, Rambaut A (2006). Relaxed phylogenetics and dating with confidence. PLoS Biology.

[PLU050C14] Edwards D, Horn A, Taylor D, Savolainen V, Hawkins JA (2008). DNA barcoding of a large genus, *Aspalathus* L. (Fabaceae). Taxon.

[PLU050C15] Fazekas AJ, Burgess KS, Kesanakurti PR, Graham SW, Newmaster SG, Husband BC, Percy DM, Hajibabaei M, Barrett SC (2008). Multiple multilocus DNA barcodes from the plastid genome discriminate plant species equally well. PLoS ONE.

[PLU050C16] Ford CS, Ayres KL, Toomey N, Haider N, Stahl JBA, Kelly LJ, Wikström N, Hollingsworth PM, Duff RJ, Hoot SB, Cowan RS, Chase Mark W, Wilkinson MJ (2009). Selection of candidate coding DNA barcoding regions for use on land plants. Botanical Journal of the Linnean Society.

[PLU050C17] Gonzalez MA, Baraloto C, Engel J, Mori SA, Pe P, The C, Rie B, González MA, Petronelli P, Riera B, Roger A, Thebaud C, Chave J (2009). Identification of Amazonian trees with DNA barcodes. PLoS ONE.

[PLU050C18] Jaén-Molina R, Marrero-Rodríguez A, Reyes-Betancort JA, Suárez JN, Caujape-Castells J, Santos-Guerra A, Ramírez Sanz L, Asensio Nistal B (2010). La flora endémica del parque nacional de Garajonay bajo la perspectiva molecular: Las secuencias de ADN como herramienta en la identificación taxonómica. Naturaleza y Parques Nacionales. Serie Investigación en la red.

[PLU050C19] Kane N, Saemundur S, Hannes D, Yang JY, Zhang D, Engels JM, Cronk Q (2012). Ultra-barcoding in cacao (*Theobroma* spp.; Malvaceae) using whole chloroplast genomes and nuclear ribosomal DNA. American Journal of Botany.

[PLU050C20] Kim SC, McGowen MR, Lubinsky P, Barber JC, Mort Mark E, Santos-Guerra A (2008). Timing and tempo of early and successive adaptive radiations in Macaronesia. PLoS ONE.

[PLU050C21] Kondo K, Shiba M, Yamaji H, Morota T, Zhengmin C, Huixia P, Shoyama Y (2007). Species identification of licorice using nrDNA and cpDNA genetic markers. Biological & Pharmaceutical Bulletin.

[PLU050C22] Kress WJ, Erickson DL (2007). A two-locus global DNA barcode for land plants: the coding *rbcL* gene complements the non-coding *trnH-psbA* spacer region. PLoS ONE.

[PLU050C23] Kress WJ, Wurdack KJ, Zimmer EA, Weigt LA, Janzen DH (2005). Use of DNA barcodes to identify flowering plants. Proceedings of the National Academy of Sciences of the USA.

[PLU050C24] Kress WJ, Erickson DL, Jones FA, Swenson NG, Perez R, Sanjur O, Bermingham E (2009). Plant DNA barcodes and a community phylogeny of a tropical forest dynamics plot in Panama. Proceedings of the National Academy of Sciences of the USA.

[PLU050C25] Lahaye R, Van Der Bank M, Bogarin D, Warner J, Pupulin F, Gigot G, Maurin O, Duthoit S, Barraclough TG, Savolainen V (2008). DNA barcoding the floras of biodiversity hotspots. Proceedings of the National Academy of Sciences of the USA.

[PLU050C26] Liu J, Möller M, Gao L, Zhang D, Zhuli D (2010). DNA barcoding for the discrimination of Eurasian yews (*Taxus* L., Taxaceae) and the discovery of cryptic species. Molecular Ecology Resources.

[PLU050C27] Maia VH, Mata CS, Franco LO, Cardoso MA, Cardoso SRS, Hemerly AS, Gomes Ferreira PC (2012). DNA barcoding Bromeliaceae: achievements and pitfalls. PLoS ONE.

[PLU050C28] Martín JL, Arechavaleta M, Borges PA, Faria B (2008). Top 100. Las 100 especies amenazadas prioritarias de gestión en la región europea biogeográfica de la Macaronesia.

[PLU050C29] Miller D, Bohs ML, Spooner DM (2009). DNA barcoding will frequently fail in complicated groups: an example in wild potatoes. American Journal of Botany.

[PLU050C30] Mort ME, Crawford DJ, Archibald JK, O'Leary TR, Santos-Guerra A (2010). Plant DNA barcoding: a test using Macaronesian taxa of *Tolpis* (Asteraceae). Taxon.

[PLU050C31] Newmaster SG, Ragupathy S (2009). Testing plant barcoding in a sister species complex of pantropical *Acacia* (Mimosideae, Fabaceae). Molecular Ecology Resources.

[PLU050C32] Newmaster SG, Fazekas AJ, Steeves R, Janovec J (2008). Testing candidate plant barcode regions in the Myristicaceae. Molecular Ecology Resources.

[PLU050C33] Ojeda DI, Santos-Guerra A, Oliva-Tejera F, Valido A, Xue X, Marrero A, Caujapé-Castells J, Cronk QCB (2013). Bird-pollinated Macaronesian *Lotus* (Leguminosae) evolved within a group of entomophilous ancestors with post-anthesis flower colour change. Perspectives in Plant Ecology Evolution and Systematics.

[PLU050C34] Ojeda I, Santos-Guerra A, Jaén-Molina R, Oliva-Tejera F, Caujapé-Castells J, Cronk Q (2012). The origin of bird pollination in Macaronesian *Lotus* (Loteae, Leguminosae). Molecular Phylogenetics and Evolution.

[PLU050C35] Oliva-Tejera F, Caujapé-Castells J, Naranjo-Suárez J, Navarro-Déniz J, Acebes-Ginovés JR, Bramwell D (2005). Population genetic differentiation in taxa of *Lotus* (Fabaceae: Loteae) endemic to the Gran Canarian pine forest. Heredity.

[PLU050C36] Oliva-Tejera F, Caujapé-Castells J, Navarro-Déniz J, Reyes-Betancort A, Scholz S, Baccarani-Rosas M, Cabrera-García N (2006). Patterns of genetic divergence on three Canarian endemic *Lotus* (Fabaceae): implications for the conservation of the endangered *L. kunkelii*. American Journal of Botany.

[PLU050C37] Pillon Y, Johansen J, Sakishima T, Chamala S, Barbazuk WB, Roalson EH, Price DK, Stacy EA (2013). Potential use of low-copy nuclear genes in DNA barcoding: a comparison with plastid genes in two Hawaiian plant radiations. BMC Evolutionary Biology.

[PLU050C38] Rambaut A (2002). SE-AL sequence alignment editor.

[PLU050C39] Saarela J, Sokoloff P, Gillespie L, Consaul L, Bull R (2013). DNA barcoding the Canadian arctic flora: core plastid barcodes (*rbcL* + *matK*) for 490 vascular plant species. PLoS ONE.

[PLU050C40] Sandral G, Remizova MV, Sokoloff DD (2006). A taxonomic survey of *Lotus* section *Pedrosia* (Leguminose, Loteae). Wulfenia.

[PLU050C41] Sass C, Little DP, Stevenson DW, Specht CD (2007). DNA barcoding in the cycadales: testing the potential of proposed barcoding markers for species identification of cycads. PLoS ONE.

[PLU050C42] Seberg O, Petersen G (2009). How many loci does it take to DNA barcode a crocus. PLoS ONE.

[PLU050C43] Song J, Yao H, Li Y, Li X, Lin Y, Liu C, Han J, Xie C, Chen S (2009). Authentication of the family Polygonaceae in Chinese pharmacopoeia by DNA barcoding technique. Journal of Ethnopharmacology.

[PLU050C44] Starr JR, Naczi RFC, Chounibard BN (2009). Plant DNA barcodes and species resolution in sedges (*Carex*, Cyperaceae). Molecular Ecology Resources.

[PLU050C45] Swofford DL (2001). PAUP* 4.0: phylogenetic analysis using parsimony (* and other methods).

[PLU050C46] VV.AA. (2000). Lista Roja de Flora Vascular Española (valoracion segun categorias UICN). Conservación Vegetal.

[PLU050C47] Wang W, Wu Y, Yan Y, Ermakova M, Kerstetter R, Messing J (2010). DNA barcoding of the Lemnaceae, a family of aquatic monocots. BMC Plant Biology.

[PLU050C48] Xiang XG, Hu H, Wang W, Jin XH (2011). DNA barcoding of the recently evolved genus *Holcoglossum* (Orchidaceae: Aeridinae): a test of DNA barcode candidates. Molecular Ecology Resources.

[PLU050C49] Yesson C, Bárcenas RT, Hernández HM, De La Luz Ruiz-Maqueda M, Prado A, Rodríguez VM, Hawkins JA (2011). DNA barcodes for Mexican Cactaceae, plants under pressure from wild collecting. Molecular Ecology Resources.

[PLU050C50] Zhang C-Y, Wang F-Y, Yan H-F, Hao G, Hu C-M, Ge X-J (2011). Testing DNA barcoding in closely related groups of *Lysimachia* L. (Myrsinaceae). Molecular Ecology Resources.

