# Supplementary material for: DNA barcodes successfully identified Macaronesian Lotus (Leguminosae) species within early diverged lineages of Cape Verde and mainland Africa
Source: AoB Plants. 2014 Aug 21;6:plu050. doi: 10.1093/aobpla/plu050 (PMC4168286; doi:10.1093/aobpla/plu050)
Supplement: Additional Information [file supp_6_plu050_index.html]

DNA barcodes successfully identified Macaronesian Lotus (Leguminosae) species within early diverged lineages of Cape Verde and mainland Africa — DNA barcodes successfully identified Macaronesian Lotus (Leguminosae) species within early diverged lineages of Cape Verde and mainland Africa — Additional Information 

# DNA barcodes successfully identified Macaronesian *Lotus* (Leguminosae) species within early diverged lineages of Cape Verde and mainland Africa

## Additional Information

Additional Information

**Files in this Data Supplement:**

- Additional Information - doc file
