# Supplementary material for: DNA barcodes successfully identified Macaronesian Lotus (Leguminosae) species within early diverged lineages of Cape Verde and mainland Africa
Source: AoB Plants. 2014 Aug 21;6:plu050. doi: 10.1093/aobpla/plu050 (PMC4168286; doi:10.1093/aobpla/plu050)
Supplement: Additional Information [file supp_plu050_plu050supp.doc]

**Table 1**

| **Taxon** | **Collection information** | **Voucher, herbarium** |
| --- | --- | --- |
| **Outgroup** |  |  |
| *Lotus japonicus* MG20 [(Regel) K. Larsen](http://www.tropicos.org/Name/13044155?projectid=0) | Cultivated from seeds at UBC | *Ojeda 69*/UBC |
| *Lotus japonicus* Gifu B-129 [(Regel) K. Larsen](http://www.tropicos.org/Name/13044155?projectid=0) | Cultivated from seeds at UBC | *Ojeda 70*/UBC |
| *Lotus filicaulis* Durieu | Cultivated from seeds at UBC | *Ojeda 71*/UBC |
| *Lotus corniculatus* L. | Vancouver, BC | *Ojeda 46*/UBC |
| *Lotus burttii* Borsos | Cultivated from seeds at UBC from Univ. Miyazaki | *Ojeda 72*/UBC |
|  |  |  |
| **Ingroup** |  |  |
| ***Lotus* section *Pedrosia* (Lowe) Christ.** |  |  |
| *Lotus arborescens* Lowe ex Cout. | Cultivated JBCVC # 164/06 Sao Nicolao CV | *Ojeda 180*/ UBC |
| *Lotus arenarius* Brot. | Cultivated UBC # PI 631779, Casablanca, Morocco  Cultivated UBC # PI 631956, Kourigba, Morocco | *-*  *Ojeda 78*/UBC |
| *Lotus arinagensis* Brawm. | DNA bank # 651 Barranco Viejo, Arinaga, GC  DNA bank # 652 Ctra faro, Arinaga, GC | José Cruz & Alicia Roca  Felicia Oliva & José Naranjo |
| *Lotus argyrodes* R.P Murray | Cult. JCVC # 5435/UDH/07 Punta de Pargo, M  Voucher, Punta San Lorenzo, M | *Ojeda 189*/UBC  ORT # 37806 |
| *Lotus assakensis* Brand | Voucher, Tarfaya-Tan Tan Sahara, Africa  DNA Bank # 1084 Tiznir, Aglou Plage, Morocco | Molero 1992 (Fernández Casas 13699)  - |
| *Lotus azoricus* P. W. Ball | Cultivated JAO # 161-00, Azores | ORT # 36336 |
| *Lotus bollei* | Cultivated JBCVC # 163/06 Sao Vicente, CV | *Ojeda 182*/UBC |
| *Lotus brunneri* Webb in Hooker | Cultivated JBCVC # 514B/07 Sal, CV | *Ojeda 181*/UBC |
| *Lotus callis-viridis* Bramwell & D.H. Davis | DNA Bank # 654 Andén Verde, GC  Andén Verde, GC | Alicia Roca & Bernardo Navarro  *Ojeda 169*/UBC |
| *Lotus campylocladus* Webb & Berthel. | Road to Cañada Teide, T  Arona-Ifonche, T | *Ojeda 206*/UBC  *Ojeda 210*/UBC |
| *Lotus creticus* L. | Cultivated JBCVC # 64/05  Cultivated UBC, PI 505409, Spain | *Ojeda 188*/UBC  *Ojeda 242*/UBC |
| *Lotus dumetorum* Webb ex R. P. Murray | Mirador Jardina, Mercedes, Anaga, T  Teno Alto, Teno, T | *Ojeda 213*/UBC  *Ojeda 228*/UBC |
| *Lotus emeroides* R. P. Murray | Inchereda, G  Epina, G | *Ojeda 207*/UBC  *Ojeda 209*/UBC |
| *Lotus eriosolen* (Maire) Mader & Podlech | Cultivated UBC # PI 631959, Ouarzate, Morocco  Cultivated UBC # PI 631784, Tiznir, Morocco | *Ojeda 244*/UBC  *Ojeda 243*/UBC |
| *L. erythrorhyzus* Bolle | Fuerteventura | A. Santos |
| *L. genistoides (nom. nudum)* | DNA Bank # 655 Cañadón Sombrío, GC | Felicia Oliva & José Naranjo/JBCVC |
| *Lotus glaucus* Sol. | Cult. JBCVC # 223/B/07 Porto Nurbita (??), M  Cult. JAO 19-05 | *Ojeda 187*/UBC  *Ojeda 233*/UBC |
| *Lotus hillebrandii* Christ | DNA Bank # 656 Llanos Chozas, P  Mirador Isora, H | José Naranjo & Paloma Maya*Ojeda 198*/UBC |
| *Lotus holosericeus* Webb & Berthel. | DNA Bank # 657 Pilancones, GC | F. Oliva, J. Naranjo, J. Navarro, I. Santana & B. Vilches/JBCVC |
| *Lotus jacobaeus* L. | DNA Bank # 658 Bordeira bei Piorno Fogo CV  DNA Bank # 2089 Ribeira Monte espia Fogo CV | Marrero et al/JBCVC  Marrero et al/JBCVC |
| *Lotus jolyi* Battand. | Voucher, Province Tan Tan, Morocco  Voucher, Province Guelmin, Morocco | *S.L. Jury & T.M. Upson 20503*/RNG  *S.L. Jury & T.M. Upson 20480*/RNG |
| *Lotus kunkelii* (Esteve) Bramwell & D. H. Davis | DNA Bank # 3805, Barranco Jinamar, GC  Cult. JBCVC # 217/07 | José Cruz & Miguel Alemán  *Ojeda 176*/UBC |
| *Lotus lancerottensis* Webb & Berth. | DNA Bank # 3823 Villaverde, Betancuira, F  Voucher, L | -  ORT # 36458 |
| *Lotus latifolius* Brand | DNA bank # 1812 Crtra. Porto Novo, CV | Marrero et al/JBCVC |
| *Lotus leptophyllus* (Lowe) K. Larsen | Barranco Guayedra, GC  GC | *Ojeda 170*/UBC  A. Santos |
| *Lotus macranthus* Lowe | Voucher, Pico Branco, Porto Santo, M  Voucher, M | ORT # 33596  ORT # 36675 |
| *Lotus maroccanus* Ball | Voucher, Talouine, Morocco  Voucher, Marrakech, Morocco | *S.L. Jury 14471*/RNG  *Fernández Casas 13737*/RNG |
| *Lotus mascaensis* Burchard | DNA Bank # 659, Cultivated JBCVC  Valle de Masca, T | José Cruz & Ruth Jaén/JBCVC  *Ojeda 200*/UBC |
| *Lotus pseudocreticus* Maire, Weiller & Wilczek | Voucher, SW Agadir Morocco  Voucher, Tamri Agadir, Morocco | *F. Damblon 84/40*/RNG  *Davies 53484*/RNG |
| *Lotus purpureus* Webb | Cult. JAO # 130-99 | ORT # 36670 |
| *Lotus salvagensis* R.P. Murray | Voucher, Salvage Grande, SGVoucher, Acantilados del NE, Salvage Islands | ORT # 35118  ORT # 35116 |
| *Lotus sessilifolius* D.C. subsp. *villossisimus* (Pitard) Sandral & Sokoloff | Las Playas, S from Parador, H | *Ojeda 196*/UBC |
| *Lotus sessilifolius* D.C. subsp. *sessilifolius* | Poris de Abona, T  Puntallana, G  Playa Pocito, Mazo, P | *Ojeda 225*/UBC  *Ojeda 208*/UBC  A. Santos |
| *Lotus* *sessilifolius* DC. var. *pentaphyllus* (Link) D. H. Davis | San Juan-Guía de Isora, T | *Ojeda 205*/UBC |
| *Lotus spartioides* Webb & Berthel. | Tamadaba, GC  Pinar Pajonales, GC  DNA bank # 662 Chira-Pinar Santiago, GC  Presa las Niñas, GC | *Ojeda 217*/UBC  *Ojeda 216*/UBC  F. Oliva, J. Naranjo, J. Navarro, I. Santana & B. Vilches/JBCVC  *Ojeda 211*/UBC |
| *Lotus tenellus* (R. Lowe) Sandral, Santos & D.D. Sokoloff | Garachico, Ermita San Roque, T | *Ojeda 446/*UBC |
| *Lotus* sp. nov. ined. 1 | Punta Hidalgo, T  South Roque Dos hermanos, Anaga, T  Teno Alto, T | *-*  *Ojeda 193*/UBC  *Ojeda 194*/UBC |
| *Lotus* sp. nov. ined. 2 (*L. leptophyllus* group) | Punta Góngora, GC | *Ojeda 167*/UBC |
| *Lotus* sp. nov. ined*.* 3 (*L. spartioides* group)  *Lotus* sp. nov. ined. 4 (*L. sessilifolius* group) | Cortijo de San Ignacio, GC  Punta Teno, Teno, T | *Ojeda 203*/UBC  *Ojeda 230*/UBC |
| ***Lotus* section *Rhyncholotus* (Monod) D.D. Sokoloff** |  |  |
| *L. berthelotii* Masf. var. *berthelotii* | Ifonche, T  Cultivated UBC, commercial plant | -  *Ojeda 238*/UBC |
| *Lotus eremiticus* A. Santos | DNA Bank # 3839, cult. JBCVC (366/04), Garafia, P | Jose Cruz |
| *L. maculatus* Breitf. | DNA Bank # 660, ex horto (8/04) Puertito Sauzal, T  Cult. UBC, commercial plant | Rafael Almeida/JBCVC  *Ojeda 239* |
| *Lotus pyranthus* P. Perez | DNA Bank # 661 JBCVC 210/99  DNA Bank # 3842, Cult. Vivero Ceplam | *Ojeda 175*/UBC  - |
